# Astrocytic Glutamatergic Transmission and Its Implications in Neurodegenerative Disorders

**DOI:** 10.3390/cells11071139

**Published:** 2022-03-28

**Authors:** Sairaj Satarker, Sree Lalitha Bojja, Prasada Chowdari Gurram, Jayesh Mudgal, Devinder Arora, Madhavan Nampoothiri

**Affiliations:** 1 Department of Pharmacology, Manipal College of Pharmaceutical Sciences, Manipal Academy of Higher Education, Manipal 576104, India; satarkers@gmail.com (S.S.); lalitha.sb@manipal.edu (S.L.B.); prasadachowdarigurram@gmail.com (P.C.G.); jayesh.mudgal@manipal.edu (J.M.); 2 School of Pharmacy and Medical Sciences, Griffith University, Gold Coast, QLD 4222, Australia; d.arora@griffith.edu.au

**Keywords:** astrocyte, glutamate, neurodegenerative diseases, V-ATPases, calcium, exocytosis, cystine/glutamate antiporter, Bestrophin-1, hemichannels

## Abstract

Several neurodegenerative disorders involve impaired neurotransmission, and glutamatergic neurotransmission sets a prototypical example. Glutamate is a predominant excitatory neurotransmitter where the astrocytes play a pivotal role in maintaining the extracellular levels through release and uptake mechanisms. Astrocytes modulate calcium-mediated excitability and release several neurotransmitters and neuromodulators, including glutamate, and significantly modulate neurotransmission. Accumulating evidence supports the concept of excitotoxicity caused by astrocytic glutamatergic release in pathological conditions. Thus, the current review highlights different vesicular and non-vesicular mechanisms of astrocytic glutamate release and their implication in neurodegenerative diseases. As in presynaptic neurons, the vesicular release of astrocytic glutamate is also primarily meditated by calcium-mediated exocytosis. V-ATPase is crucial in the acidification and maintenance of the gradient that facilitates the vesicular storage of glutamate. Along with these, several other components, such as cystine/glutamate antiporter, hemichannels, BEST-1, TREK-1, purinergic receptors and so forth, also contribute to glutamate release under physiological and pathological conditions. Events of hampered glutamate uptake could promote inflamed astrocytes to trigger repetitive release of glutamate. This could be favorable towards the development and worsening of neurodegenerative diseases. Therefore, across neurodegenerative diseases, we review the relations between defective glutamatergic signaling and astrocytic vesicular and non-vesicular events in glutamate homeostasis. The optimum regulation of astrocytic glutamatergic transmission could pave the way for the management of these diseases and add to their therapeutic value.

## 1. Introduction

**Figure 1:**
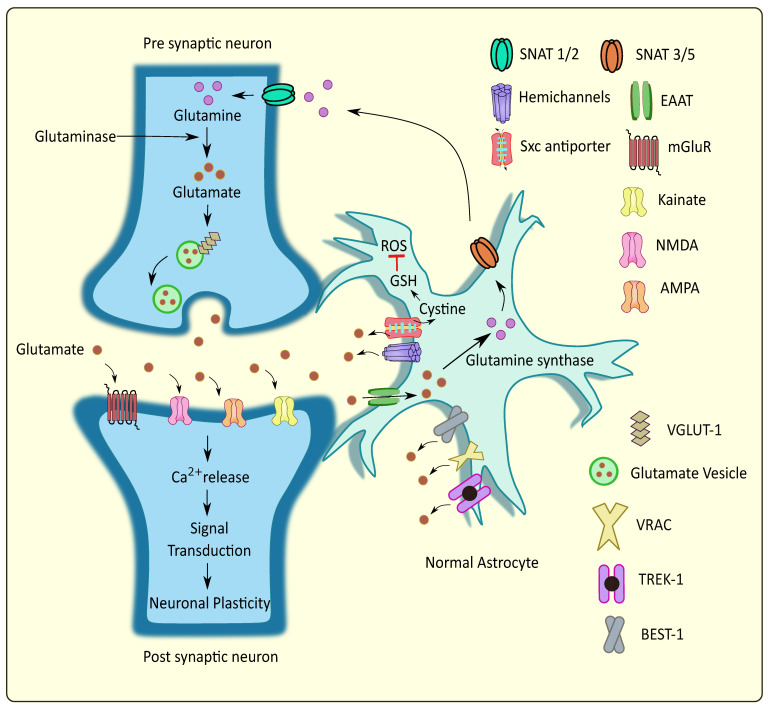
Glutamate homeostasis at the tripartite glutaminergic synapse. Glutamate is released from the synaptic vesicles into the synaptic cleft through Ca^2+^ mediated exocytosis. Upon binding to the postsynaptic receptors, their activation leads to Ca^2+^ elevations and subsequent synaptic plasticity. Neighboring astrocytes mediate glutamate uptake and convert certain amounts to glutamine followed by its transport to the presynaptic neurons, while some of the glutamate is released into the extracellular regions through various pathways. This regulates the glutamate homeostasis at the tripartite glutamatergic synapse. SNAT: sodium-coupled neutral amino acid transporter; Sxc antiporter: cystine/glutamate antiporter system xc; VGLUT: vesicular glutamate transporter; VRAC: volume regulated anion channels; TREK: TWIK related potassium channel; BEST: bestrophin; ROS: reactive oxygen species; GSH: glutathione.

Astrocytes are the specialized glial cells that represent the majority of non-neuronal cells present in large numbers in the central nervous system (CNS). They serve as a major source of energy for neurons and control neuronal excitability by maintaining ion and neurotransmitter homeostasis [1]. They provide structural support to axonal bundles in neuronal cells along with the required metabolic support [2]. Further, astrocytes are directly involved in neuronal signaling at the tripartite synapse [3]. They are endowed with the machinery of various receptors involved in neurotransmission, along with transporters that facilitate responses to the neuronal signals. Studies indicate that astrocytes react to hormones, neurotransmitters and other stimuli, and possibly contribute, but not always, to the elevation in cytosolic calcium (Ca^2+^) levels, with the subsequent release of gliotransmitters [4]. Gliotransmitters involve the classical neurotransmitters such as glutamate and γ aminobutyric acid (GABA), Adenosine triphosphate (ATP), neurosteroids and inflammatory mediators, which are capable of modulating synaptic transmission and plasticity. The optimum release of gliotransmitters under physiological conditions regulate synaptic transmission, cerebral blood flow, neuronal network synchrony and mediate immunoinflammatory responses in the brain [5]. Glutamate is the most prominent excitatory amino acid neurotransmitter in the CNS and mediates rapid excitatory transmission by activating both ionotropic receptors such as NMDA (N-methyl-D-aspartate), AMPA (α-amino-3-hydroxy-5-methyl-4-isoxazole propionic acid) and Kainate receptors; and metabotropic glutamate receptors (mGluRs). Astrocytes play a pivotal role in maintaining the extracellular glutamate levels [6]. It is present in micromolar concentrations at the synaptic cleft, which is maintained by the astrocytes and neuronal reuptake mechanisms [7]. The glutamatergic synapse is ensheathed by astrocytes expressing high levels of glutamate reuptake transporters, excitatory amino acid transporter-1 (EAAT-1), and EAAT-2. Glutamate–aspartate transporter (GLAST) and Glutamine transporter-1 (GLT-1) are homologous rodent terms used for EAAT1 and EAAT2 respectively [8]. About 90% of glutamate present in the synaptic cleft is withdrawn by the astrocytes through the highly efficient EAAT-1 transporters which prevent glutamate accumulation and excitotoxicity as shown in Figure 1 [9,10]. This is one of the major reasons for the maintenance of low synaptic concentrations of glutamate in the submicromolar to nanomolar range [11]. However, Herman et al. reported no significant differences in the concentration gradients of glutamate in synaptic and extrasynaptic regions [12]. Glutamate excitotoxicity occurs due to elevated synaptic glutamate concentration attributed to factors such as excessive glutamate release from neurons and glial cells, impaired clearance, enhanced glutamate receptor sensitivity, compromised postsynaptic neurons, excessive Ca^2+^ concentrations, and so forth, which ultimately lead to cell death [13,14,15]. Such glutamate-mediated toxicity has been linked to several neurological disorders such as Alzheimer’s disease (AD), Parkinson’s disease (PD), Amyotrophic lateral sclerosis (ALS), and epilepsy [5,6,7,8].

In the pathogenesis of AD, the Aβ 1–42 induces glutamate toxicity due to altered glutamate reuptake from the synaptic cleft as a result of reduced astrocytic GLT-1 [16]. In a transgenic rodent model of PD, a mutant α-synuclein caused the aggregation of α synuclein in the astrocytes and along with severe astrogliosis, thereby down-regulating glutamate transporters which resulted in microglia activation and cytokine overproduction [17]. Similarly, in the condition of ALS, GLT-1 was downregulated and its levels were influenced by components like tumor necrosis factor α (TNF-α), nuclear factor kappa B (NFκB) signaling, upregulation of astrocyte elevated gene–1 (AEG-1) andthe knocking out of membralin, an important component of endoplasmic reticulum (ER) [18,19]. With the astrocytic dysfunction and defective reuptake transporters significantly contributing to excitotoxicity in neurons in neurodegenerative disorders, the implications of vesicular and non-vesicular release mechanisms of glutamate found in astrocytes remain poorly understood. Therefore, the current review corroborates the importance of glutamate release and uptake mechanisms of astrocytes, focusing on neurodegeneration. 

## 2. Major Participants in Astrocytic Glutamatergic Transmission and Their Association in Neurodegenerative Disorders

### 2.1. Calcium Mediated Exocytosis

Astrocytes are essential in modulating neuronal activity and synaptic neurotransmission [20]. Nerve terminals are encased with astrocytes and are strategically located to communicate effectively with synapses [20]. The astrocytic responses towards synaptic stimulation have been well established [21]. They mediate Ca^2+^ dependent glutamate release and regulate synaptic neurotransmission [22]. As the intracellular Ca^2+^ level required for astrocytic glutamate release is within physiological limits, this release can be exploited as a signaling mechanism to alter synaptic neurotransmission and plasticity within the CNS [23]. Primarily, the generation of intercellular Ca^2+^ waves (ICW) involves the release of Ca^2+^ from the ER via G protein coupled receptor (GPCR) activation [24]. Studies on glia revealed the presence of mGluRs which, upon activation by physiological ligands, resulted in the synthesis of inositol 1,4,5-triphosphate (IP3) and subsequent Ca^2+^ release [25]. This release of Ca^2+^ promoted the onset and maintenance of ICW of the glial cell, which provided long-range signaling [26]. These waves depict the rise in Ca^2+^ levels in the cytoplasm, which communicates with other cells and has a wave-like appearance that radiates from its originating source. The ICW is initiated by the release of ATP that follows after hemichannel opening [27]. Interestingly, it was demonstrated that glutamate concentration was directly proportional to the frequency oscillations of Ca^2+^ waves [28]. This Ca^2+^ is released by the hippocampal astrocytic cells from intracellular storage both naturally and in response to the activation of Gq-linked GPCR by binding IP3 to its receptor (IP3R). The released Ca^2+^ in astrocytes is essential and sufficient for the secretion of gliotransmitters, such as ATP and glutamate, and further affects the neuronal activity. IP3R type 2 (IP3R2) appears to be the major IP3R expressed by astrocytes [29]. IP3R-mediated Ca^2+^ signaling is speculated to cause the activity-dependent and selective release of chemical transmitters. In astrocytes, IP3R2 was once the only known Ca^2+^ channel; however, Ca^2+^ imaging techniques have recently determined new Ca^2+^ sources including mitochondria [30]. Rakers et al. reported that the release of IP3R2-dependent Ca^2+^ from internal reserves causes a rise in astroglial Ca^2+^ during neurological disorders including stroke [31]. Astrocytes emit several signaling chemicals such as glutamate, D-serine and ATP. Activities of these molecules, such as modulating synaptic transmission and influencing particular behavior, are vigorously studied, but the identity of their cellular compartments remains unknown [32]. The pharmacological inhibition of vesicular glutamate transporters (VGLUTs) significantly decreased exocytotic glutamate release from astrocytes which is a Ca^2+^ dependent phenomenon, indicating that these transporters may be instrumental in the astrocytic glutamate release in CNS. VGLUTs transfer cytoplasmic glutamate into exocytotic vesicles, which are propelled by a proton gradient created by vacuolar ATPases (V-ATPases) [33]. VGLUT1 and 2 are also seen, along with synaptic-like vesicles [34]. Montana et al. demonstrated that VGLUTs 1 and 2 are present in rat astrocytes and show high immunoreactivity, justifying their role in the glutamate release via exocytosis [35]. Conversely, the VGLUT mRNAs were absent in astrocytic transcriptome [36]. This supported the notion made by Li et al. that VGLUTs were absent in the astrocytes [37]. The cytosol of astrocytes have high glutamate concentrations ranging from 0.1–5 mM, but extracellular glutamate levels lie within the sub-micromolar range [38]. In astrocytes, glutamate is packed into synaptic-like vesicles and is released in a Ca^2+^ dependent mechanism, demonstrating the contribution of astrocytes in glutamatergic transmission.

**Figure 2:**
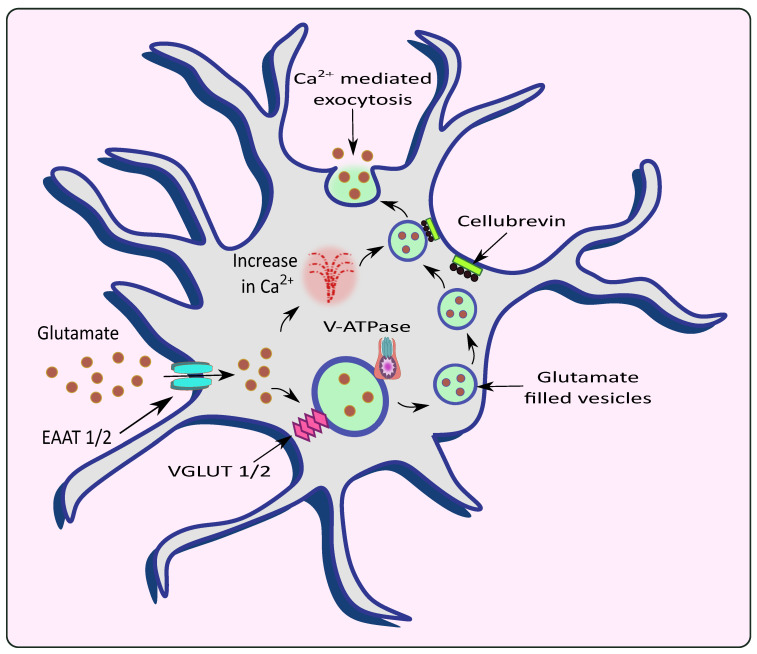
Release of glutamate from astrocytes via Ca^2+^ mediated exocytosis. Astrocytic EAAT promotes the uptake of glutamate from the synaptic cleft which is filled into the vesicles by VGLUT in the presence of V-ATPase. The rise in intracellular Ca^2+^ causes the vesicles to fuse with the membrane with the help of an astrocytic vesicular SNARE protein cellubrevin and promotes Ca^2+^ mediated exocytosis of glutamate.

Astrocytes exert controlled glutamate exocytosis via a protein complex called the soluble N-ethylmaleimide-sensitive factor (NSF) attachment protein (SNAP) receptor (SNARE) complex, which regulates vesicle fusion [39]. Synaptobrevin 2, Syntaxin 1, and synaptosome-associated protein of 23 kDa are all part of the core SNARE complex (SNAP-23), while Synaptotagmin 4 is a Ca^2+^ sensor. SNARE proteins are found on both the vesicular membranes and the presynaptic plasma membranes that cause membrane fusion [40]. In the neurons, the vesicle-associated membrane protein 2 (VAMP2) binds to Syntaxin and synaptosomal-associated protein 25 (SNAP25) on the cell membrane to form the SNARE complex. Synaptotagmin 1, a Ca^2+^ sensor expressed by neurons, detects the Ca^2+^ rise caused by Ca^2+^ entry via voltage-gated Ca^2+^ channels and triggers the fusion of vesicles to the cell membrane, releasing glutamate. VAMP2/VAMP3, Syntaxin and SNAP25/SNAP23 are all expressed by astrocytes with similar functions [41]. However, Bezzi et al. suggested that, instead of VAMPs, astrocytes express cellubrevin—a SNARE complex of astrocytic vesicles [34]. Further, research revealed that astrocytes produce Synaptotagmins 4, 7, and 11, which cause the release of glutamate from vesicles in response to a rise in intracellular Ca^2+^ levels in similar fashion to neurons. Intracellular Ca^2+^ levels need to rise in the range of 250 to 350nM to stimulate astrocytic glutamate release [41,42,43]. With an increase in Ca^2+^ levels, the vesicles fuse with SNARE proteins and undergo Ca^2+^-mediated exocytosis to release glutamate [44,45] as shown in Figure 2. Increasing Ca^2+^ concentrations by overstimulation of glutamate receptors could lead to excitotoxicity and neuronal death [46]. Therefore, impairment in Ca^2+^ signaling could lead to the progression or worsening of neurodegenerative diseases such as AD and PD.

AD is associated with progressive neurodegeneration and marks its presence primarily through cognitive deficits in patients. Its hallmarks, namely neurofibrillary tangles (NFTs) and the occurrence of amyloid-beta (Aβ) plaques, have been blamed for the progression and worsening of AD [47]. Mostly, it is believed that AD pathology occurs through the amyloid cascade hypothesis originating via the amyloid precursor protein (APP). The action of enzymes namely β secretases and γ secretases produce insoluble Aβ that confers neurotoxicity [48]. Epigenetic modification, proteolysis abnormalities, oxidative stress, neuroinflammation, hampered mitochondrial function, and faulty autophagy are some of the variables that contribute to accelerated aging and neurodegenerative disorders [49,50,51,52]. However, in recent years, the pathology of AD has expanded in multiple dimensions including the pathogenic role of dysfunctional glial cells and the excessive release of neurotransmitters such as glutamate [53]. 

**Figure 3:**
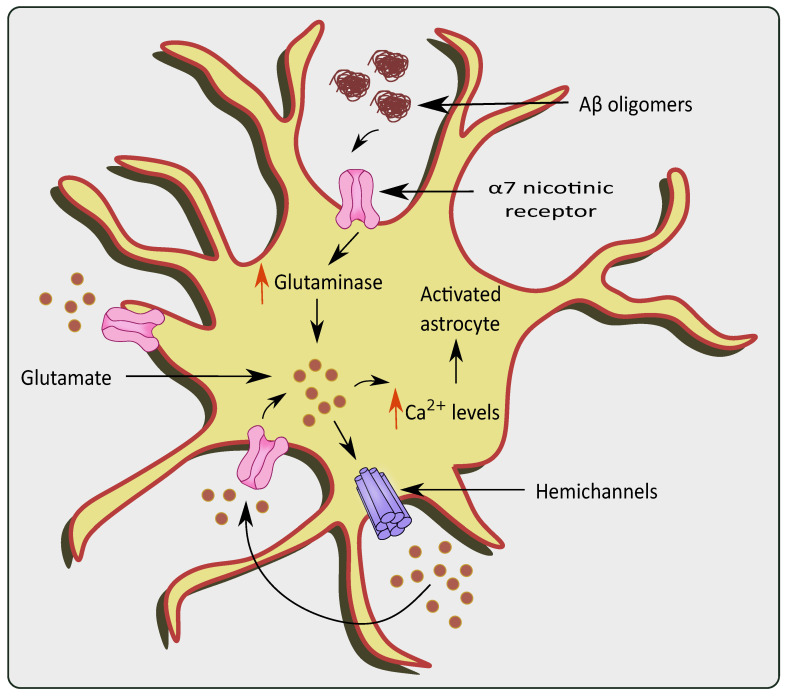
Glutamate excitotoxicity via overstimulation of α 7 nicotinic receptors in the presence of AD pathologies. The presence of Aβ oligomers causes activation and over-stimulation of α 7nAChRs, which increase levels of glutaminase and glutamine in the astrocytes. This causes a rise in Ca^2+^ levels, ultimately stimulating hemichannels to release more glutamate causing glutamate excitotoxicity.

Neurotoxicity occurs due to an over-accumulation of extracellular glutamate during Aβ aggregation [54,55]. The effect of Aβ 1–42 on a α7 subunit containing nicotinic receptors (7nAChR) could also increase internal Ca^2+^ currents and subsequent glutamate uptake/release causing glutamate excitotoxicity as shown in Figure 3 [56]. Similarly, Aβ 1–42 has picomolar affinity for the 7nAChR, which is known to enhance glutamate release when activated [57,58]. Lower levels of endogenous Aβ 1–42 are necessary for normal brain function, while at a higher concentration the resultant accumulation and aggregation results in neurotoxicity [59] Through the (7nAChR), Aβ 1–42 can cause glutamate release in the hippocampal nucleus, which is cleared from the extracellular space quickly (msec) by high-affinity EAATs [60,61]. Nicotine-induced glutamate release via the 7nAChR is supported by the hypothesis that Aβ 1–42 binding near the nicotinic site on the 7nAChR can elicit glutamate release [58]. Furthermore, increased Aβ 1–42 synthesis proportionately increases glutamate release in the Cornu Ammonis (CA) 1 area of the amyloid precursor protein/Presenilin 1 (APP/PS1) mouse model [62]. Lower density of 7nAChRs in the CA3 region could explain why an elevated Aβ 1–42 concentration is required to elicit greater glutamate release in the hippocampal region. Aβ 1–42 protein deposition has been reported to occur initially in the CA1 and DG, followed by the CA3 in patients with AD [63]. Hascup and colleagues discovered that enhanced Aβ 1–42 evoked glutamate release in the CA1 and DG at lower doses [64]. The presence of Aβ causes the activation of 7nAChRs present in the astrocytes of the hippocampal regions [65]. Similarly, 7nAChR over expression was observed in the rat astrocytes in the presence of AD pathogenesis [66]. It is evident that 7nAChRs elevate the intracellular Ca^2+^ levels by stimulating Ca^2+^ release from intracellular reserves of astrocytes [67]. 

Similarly, the actions of Aβ 25–35 on the astrocytic purinergic receptors promote Ca^2+^ level elevation [68]. Chronic calciumopathy, observed in AD, affects neuronal Ca^2+^ homeostasis and Ca^2+^ signaling [69]. In AD, the senile plaques promote Ca^2+^ hyperactivity in astrocytes that trigger excessive glutamate release. The released Ca^2+^ from the endoplasmic reticulum is essential for astrogliotic response. Therefore this higher Ca^2+^ signaling, as seen in the entorhinal region and prefrontal cortices of rodent AD models, suggests the abnormalities associated with these pathologies [70]. Additionally, enhanced astroglial Ca^2+^ signaling followed by glutamate excitotoxicity has been seen in mice with the APP/PS1 gene [71,72]. Recently, Pham et al. found that astrocytic Aβ exposure resulted in both Ca^2+^ dependent and independent glutamate release, which caused excitotoxicity. Interestingly, a notable amount of glutamate was released before the Ca^2+^ elevation during Aβ administration, followed by a surge in glutamate with a subsequent rise in Ca^2+^ [68].

**Figure 4:**
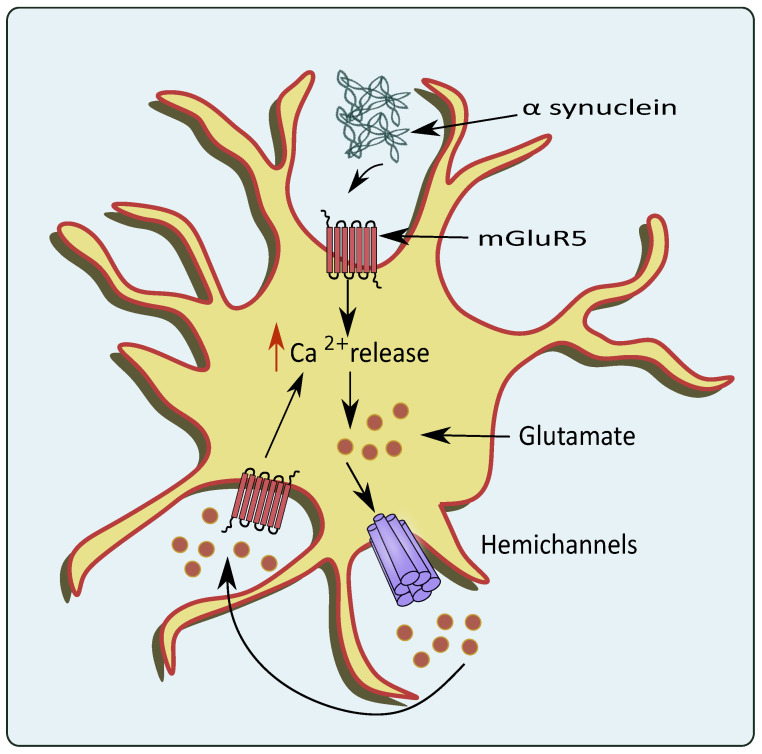
Glutamate excitotoxicity mediated through overexpression of mGluR5 by PD pathologies. In the conditions of PD, the α-synuclein activates astrocytic mGluR5 that elevates intracellular Ca^2+^ levels and stimulates the release of glutamate through the hemichannels.

PD is another neurodegenerative disease with impairment in neurons present in the dopaminergic system [73]. The region of substantia nigra pars compacta (SN) residing within the midbrain is highly affected. Pathological hallmarks such as Lewy bodies and α synuclein deposition are considered to play a role in the progression of PD [74]. Patients diagnosed with PD mainly show tremors, muscle rigidity, motor deficits, gait instability, and memory deficits [75]. Recently, autonomic dysfunction has been attributed to PD pathology [76]. Impaired homeostasis of neurotransmitters such as glutamate also plays a pivotal role in the pathogenesis of PD [77]. Inflammatory processes-induced astrocytic glutamate excitotoxicity has also been linked to PD, which causes changes in glutamate transporters and receptor expression [78,79]. Moreover, the accumulation of α-synuclein increases the Ca^2+^ depolarization-dependent release of presynaptic glutamate as demonstrated using synaptoneurosomes obtained from the forebrain [80]. The α-synuclein releases glutamate in a Ca^2+^ dependent mechanism, which further activates the extrasynaptic NMDA receptors, and causes neuronal damage [81]. This increase in glutamate activated the AMPA receptors that further upregulate the glutamate release [82]. Interestingly, α-synuclein mobilizes the glutamate vesicles from the pool of reserves [83]. mGluR5 overexcitation upon the binding of α-synuclein also stimulates the release of Ca^2+^ leading to glutamate excitotoxicity as shown in Figure 4 [84]. A small protein, DJ-1 encoded by *PARK7* gene has also been associated with PD pathogenesis, and knockout of *PARK7* hampered glutamate uptake via astrocytes. This altered glutamate uptake was associated with the downregulation of EAATs, which led to neurotoxicity in PD patients [85]. Similarly, Wang et al. showed that JWA knockout mice in pathologies of PD reduced the glutamate uptake by hampering EAAT 2 expression [86].

ALS is a neurodegenerative disease that involves the degeneration of motor neurons in the CNS [87]. Its prominent characteristic features include motor weakness and loss of motor neurons, gliosis and atrophy of skeletal muscles [88]. Significantly reduced expression of GLT-1 in the motor cortex and spinal cord has been proposed to be one of the main factors leading to glutamate excitotoxicity in ALS [19].

Metadata analysis of studies of transgenic ALS cell cultures suggests that extrinsically boosting the astrocytic GLT-1 level before ALS end-stage may improve the reuptake of glutamate and could be considered a therapeutic strategy. Excitotoxicity could be perhaps due to the ability of those ALS astrocytes that render themselves susceptible to even minor alterations in the neuronal environment. Further, at pre-onset, lowering astrocytic GluR1 levels could help to lessen intracellular Ca^2+^ [89]. Mutations in valocin containing protein (VCP) genes are causative for ALS. VCP mutant astrocytes showed reduced glutamate uptake and induced reactive astrocytes. This could serve as a protective mechanism at first but becomes toxic over time due to impaired homeostasis. These ALS astrocytes up-regulate inflammatory signaling and are seen to reduce its supportive actions to neurons [90]. 

In a mouse model of ALS, an increased influx of Ca^2+^ has been shown to be instrumental in glutamate exocytosis by affecting vesicle fusion and release mechanisms [91]. Furthermore, SOD1 gene mutations in familial ALS interfere with mitochondrial function and prevent glutamate reuptake, thus causing glutamate excitotoxicity [27]. Another interesting target seen in familial ALS is the upregulation of the ATP- binding cassette transporters (ABC) transporter glycoprotein (P-gp). It is known to be upregulated by NMDA receptors activated by glutamate that is excessively secreted by astrocytes with mutant superoxide dismutase 1 (SOD1) [92]. The sporadic and familial mice ALS models have shown a marked reduction in the GLT-1 [93]. Astrocytes that express mutated SOD1 fail to regulate the expression of glutamate receptor’s GluR2 subunit, and is present in motor neurons which leads to higher Ca^2+^ levels and motor death [94].

Parpura et al. via flash photolysis increased internal Ca^2+^ in astrocytes to monitor Ca^2+^ and glutamate levels that elicited slow inward currents. These electrophysiologically recorded signals showed that small variations in astrocytic Ca^2+^, from 84 nM to 140 nM, elicited large glutamatergic currents in adjacent neurons. Therefore, the astrocytic glutamate release pathway is activated at normal levels of internal Ca^2+^, as glutamate further elevates Ca^2+^ in astrocytes to values surpassing 1.8 μM [42]. When cytosolic Ca^2+^ levels rise, mitochondria quickly absorb Ca^2+^ to avoid Ca^2+^ overload in the cytosol. But this excessive mitochondrial Ca^2+^ uptake could lead to mitochondrial Ca^2+^ overload and result in events like increase in reactive oxidative species (ROS), glutamate production inhibition of ATP synthesis mitochondrial permeability transition pore (mPTP) opening, the release of cytochrome C, activation of caspases, and apoptosis. The rise in Ca^2+^ levels is transient in nature and occurs in the presence of disease pathologies involved in neurodegenerative diseases such as AD [42,95], PD [96], multiple sclerosis [97] and Huntington’s disease [98].

Therefore, in such pathological conditions, astrocytes respond rapidly with numerous cellular adaptations, including morphological and functional rearrangements, gene and protein expression alterations, as well as changes in its secretome, which are collectively called reactive astrogliosis [99]. The hallmark features of these reactive astrocytes are cell hypertrophy and up-regulation of Glial fibrillary acidic protein (GFAP) and vimentin (intermediate filaments). Along with these, they exhibit aberrant Ca^2+^ signaling in a spatial and temporal dependent fashion, depending on the pathological condition [100]. Since these Ca^2+^-induced events are linked with glutamate exocytosis mediated by Ca^2+^, their causal role in these types of neurodegenerative disorders needs to be emphasized. 

### 2.2. Vacuolar ATPases (V-ATPases)

The V-ATPases are proton pumps with multiple subunits composed of a peripheral component V1 attached to an internal membrane-associated component V0 [101]. The V0 is associated with the translocation of proteins while V1 is responsible for the hydrolysis of ATP [102]. These pumps operate in conjunction with eight subunits that are present in V1 and six subunits present in V0 [103]. The regulation of these pumps occurs in multiple processes such as the reversible dissociation of V1 and V0 subunits, disulfide bond formation, the altered proton transport to ATP hydrolysis ratio expressed as coupling efficiency, and modulation in the conductance of counterions [104].

**Figure 5:**
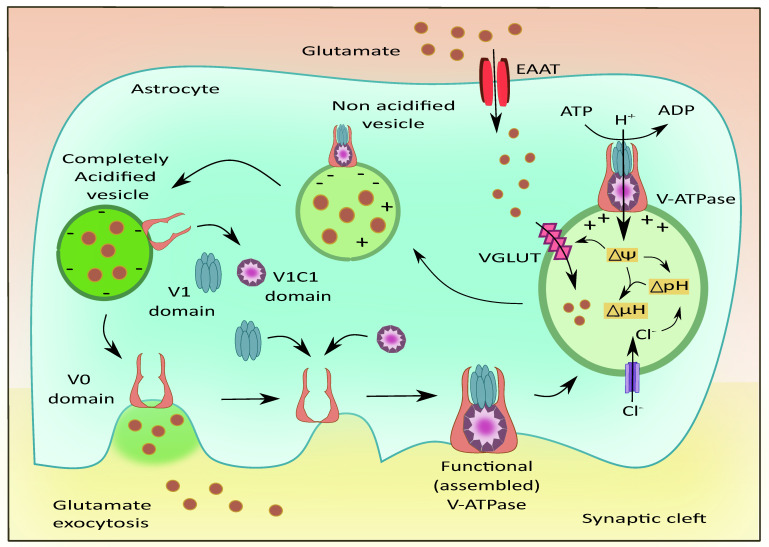
Role of V-ATPases in vesicular glutamate filling and its exocytosis. The subunits of V-ATPases namely V0, V1, and V1C1 assemble to form a functional entity that generates the membrane potential and pH gradient necessary for the uptake of glutamate into the vesicles via VGLUT. Upon complete acidification of the vesicle, the V1 and V1C1 domains detach from the complex while the V0 domain facilitates membrane binding of the vesicle followed by glutamate exocytosis.

The presence of V-ATPases has been previously identified on the plasma membrane of astrocytes [105] and presynaptic neurons; along with their vesicular membranes [106]. V-ATPases play a role in maintaining acidic pH and the membrane potential to drive the filling of the vesicles with neurotransmitters as shown in Figure 5 [107]. They are also reported to exist in G1 and G2 isoforms where the G2 isoform is instrumental in maintaining the acidification of synaptic vesicles [108]. The Voa1 and Voa2 components of V-ATPases were identified on the vesicles in PC12 cell lines [109]. Interestingly, V-ATPases were recently known to have no direct contribution to the fusion of synaptic vesicles. They are released as V0, V1, and V1C1 components from the acidified vesicles [110]. The V1 deficient synaptic vesicles bind to the plasma membrane and cause the recruitment of other components upon contact with luminal pH. This leads to endocytosis of vesicles that contain the fully assembled V-ATPases [111]. This is followed by the recruitment of H+ ions into the vesicular lumen, the generation of membrane potential, and the filling of glutamate [112]. 

The VGLUTs are responsible for recruiting glutamate molecules into the synaptic vesicles [113]. Montana et al. have reported the presence of VGLUTs in astrocytes and their role in astrocytic glutamate transmission [35]. The V-ATPases were localized on the surface of astrocytes present in the hippocampus, and these pumps were responsible for the regulation of intracellular pH (pH_i_) [114]. The primary astrocytic cultures are mainly dependent on HCO3—independent mechanisms for maintaining the pH_i_ in comparison to cultured astrocytes [115]. The process of neurotransmitter vesicular refilling mainly relies on the electrochemical gradient. This gradient is fulfilled by the V-ATPases and also by chloride ion channels by generating the required chemical gradient and membrane potential to facilitate this uptake [116]. To a small extent, glutamate uptake is dependent on the chemical gradient, while optimum membrane potential is extremely necessary for the vesicular entry of glutamate. Interestingly, since glutamate itself is anionic, it acidifies the vesicles and activates the V-ATPases, enhancing the vesicular filling of glutamate via VGLUT [117]. Another important factor for the optimum functioning of the V-ATPases is the ratio of ADP and ATP in astrocytes. Altered levels of ATP could affect the functioning of these pumps and prevent the development of the required proton gradient that would affect glutamate uptake into the vesicles thus affecting the release of glutamate [118]. The mode of ATP release could be through a Ca^2+^ dependent method—where the ATP is transported to the plasma membrane of astrocytes through secretory vesicles—and a Ca^2+^ independent method—where astrocytic hemichannels and purinergic receptors, could release ATP [119].

The blockade of astrocytic V-ATPase with bafilomycin A1, a V-ATPase inhibitor, showed alterations in the expression of TNF-α [120] and low levels of glutamate release in astrocytes [35]. Similarly, the administration of N-ethylmaleimide (NEM) reduced the pHi and incorporation of a more selective inhibitor of V-ATPases, 7-chloro-4-nitroben-2-oxa-1,3-diazole confirmed the vacuolar nature of these pumps [121]. NEM appears to activate potassium ion antiport [122] but the exact mechanism by which it reduces pH_i_ is not known. 

The lysosomal degradation is executed by the hydrolytic enzymes, which are activated in acidic pH. The V-ATPase maintains this acidic pH by pumping protons into the lumen, by utilizing ATP [32]. Lysosomal exocytotic release occurs much more slowly as compared to the release of neurotransmitters through vesicles [123]. As the lysosomes contain ATP, lysosomal impairment prevents ATP-mediated Ca^2+^ release, ultimately affecting astrocytic exocytosis governed by Ca^2+^ stimulation [124]. This provides evidence for the importance of lysosomal functioning in astrocytes.

The V-ATPases have been implicated in the pathogenesis of neurodegenerative diseases like AD and PD. As discussed earlier, the reversible translocation of V0 and V1 subunits are essential in the optimal functioning of the V-ATPases. The ATP6V1-A was found to be downregulated in the conditions of AD that impacted the neurotransmitter release from synaptic vesicles and prevented phosphorylation and phagosome formation thus worsening AD pathologies [125]. ATP6AP2 is an important accessory protein that promotes neuronal growth in the CNS. Recently, splice variants of ATP6AP2 demonstrated defects in the acidification of lysosomes and progressed towards neuronal death. In in vitro systems, the ATP6AP2 deficits led to impaired V-ATPase assembly, thus affecting its function. The loss or mutations in Presenilin1 (PS1) have contributed to the pathologies of AD [126]. This could be the reason why PS1 deletions showed impaired acidification of lysosomes and impaired autophagy, thus hampering the clearance of oligomers in AD [127].

In the conditions of PD, lysosomal clearance of aggregates, such as α-synuclein, proteins with misfolded morphology, or debris, is essential which is regulated by V-ATPase activity [128]. The mutations in the ATP6AP2 were correlated with progression towards parkinsonism in patients with X-linked parkinsonian syndrome [129]. These studies demonstrate the importance of V-ATPases and their putative implication in neurodegenerative diseases, which is still underexplored.

### 2.3. Cystine/Glutamate Antiporter System xc (Sxc)

**Figure 6:**
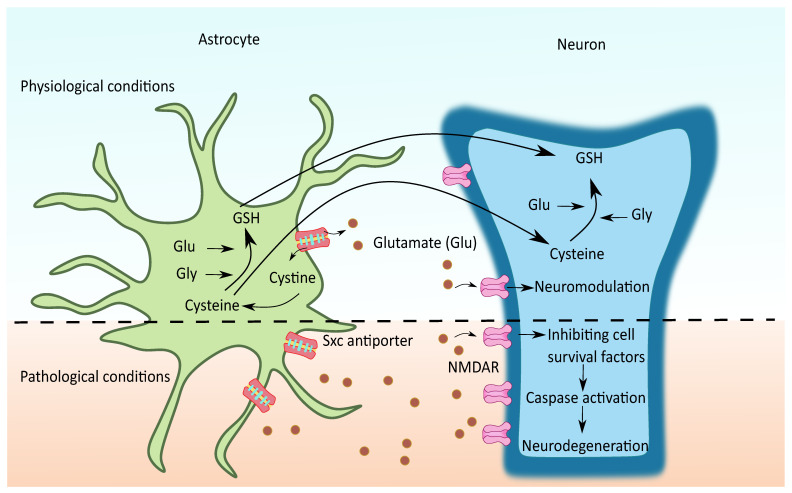
Possible role of Sxc- in neurodegenerative diseases. During physiological conditions, the cystine imported from Sxc- is rapidly reduced to cysteine, an essential substrate for glutathione (GSH) synthesis. Mostly cysteine and sometimes GSH are transported from glia to neurons to meet the neuronal demands of GSH. The non-vesicular glutamate thus released with the exchange of cystine acts over the extrasynaptic NMDAR and mGluR and modulates the synaptic plasticity. In certain conditions, such as oxidative stress, the overexpression of glial Sxc- contributes to elevated glutamate levels followed by extrasynaptic glutamate receptor activation, mediating the neurotoxicity by favoring related processes such as inhibiting cell survival factors and activating the caspases.

Sxc- is another important mechanism of astroglial glutamate release in several regions of the brain and spinal cord. It is an anionic amino acid antiporter that exports glutamate for cystine. Cystine is critical for glutathione synthesis and is needed for maintaining the cellular anti-oxidant pool. On the other hand, the released glutamate can act extrasynaptically and potentially modulate synaptic plasticity [130], as seen in Figure 6. In CNS, Sxc- has been characterized mainly in astrocytes and other sites such as microglia, immature neurons, and ependymal cells. Unlike other astrocytic glutamate release mechanisms, the physiological function of Sxc- is well known as it is an important source of glutathione [131]. It is known that astrocytes are a major supply of glutathione to neurons; Sxc- is one such principle mechanism through which astrocytes provide cysteine and other glutathione precursors to neurons [132]. Under oxidative stress or glutathione deficient conditions, the expression and activity of Sxc- is increased to combat the reactive oxygen species as a protective mechanism. On the other hand, the elevated glutamate concentrations due to the increased Sxc- might contribute to neuronal excitotoxicity in certain neurological conditions.

Thus, this transporter functions across the crossroads of oxidative stress and glutamate excitotoxicity and is therefore of significant interest in diseases where both these are implicated, such as amyotrophic lateral sclerosis (ALS), epilepsy, glioma, schizophrenia, and so forth [130,133]. However, the complete understanding of the role of the Sxc- transporter in neurological diseases is still lacking, whether its upregulation is beneficial for overcoming oxidative stress or detrimental due to its potential to lead to excitotoxicity. A detailed description of important aspects of the transporter, including the pharmacology and regulation, is available in the previous reviews [77,78]. In the scope of this review, the involvement of astrocytic Sxc- mediated glutamate release in neurodegenerative disorders is covered.

Of note, around 50–70% of extracellular glutamate was reduced in PD and epileptic mice with Sxc- knockout indicating the importance of Sxc- mediated glutamate regulation in CNS [134,135]. Possibly due to their proximal location in the astrocytes, the glutamate released by them primarily acts upon the extrasynaptic NMDA receptors in the neurons. Since the extrasynaptic regions of neurons have high-affinity glutamate receptors such as NMDA and mGLUR5, the extrasynaptic signaling can play a significant role in neuromodulation under physiological conditions [78,130]. Whereas, under pathological conditions where the glutamate reuptake transporters are dysfunctional, the non-vesicular glutamate released by Sxc- adds to the elevated extracellular glutamate levels. The glutamate released promotes extrasynaptic NMDAR activation, which preferentially activates cell death pathways leading to neurodegeneration [15].

An enormous amount of evidence exists indicating the accumulated glutamate levels and associated neurotoxicity in ALS patients. ALS is a fatal progressive motor neuron degenerative disease with a mean survival rate of 3–10 years. The etiology of almost 90–95% of cases of ALS is not known; these are grouped as sporadic ALS (sALS). The involvement of impaired homeostasis, glutamate excitotoxicity, oxidative damage, and mitochondrial dysfunction has been widely studied to be involved in motor neuron death in sALS [136]. In addition, the motor neurons have intrinsically low Ca^2+^ buffering capacity and high AMPA receptors, making them selectively susceptible to excitotoxicity [137]. Past research indicates that the reduced expression of the EAAT2/GLT-1 glutamate reuptake transporter in astrocytes of postmortem brain and spinal cord tissues of ALS is the prime reason for the elevated glutamate levels [138]. The decrease in EAAT2 expression was also seen in several other neurodegenerative disorders such as AD, Huntington’s disease, and epilepsy, corroborating the causal role of astrocytes to glutamate toxicity [139]. However, recent studies highlight the glutamate released by non-neuronal cells as an additional significant factor contributing to the increased glutamate levels in ALS [9]. This may be the reason for the insufficient action delivered by the drug, Riluzole, a neuronal glutamate release inhibitor in ALS, which is the only FDA approved drug for the treatment of ALS [140]. Further, it was observed that astrocytes present in the spinal cord of ALS patients showed substantial elevation in the Sxc- levels as compared to controls [141]. Increased Sxc- mediated glutamate release was observed even before the reduction in EAAT2, and this contributed to the early glutamate toxicity during the disease initiation in the (SOD1)-G93A transgenic rodent model of ALS [46]. A recent study showed that the deletion of xCT (core protein of Sxc-) delayed the rate of disease progression in a mutant SOD1 ALS mouse model [142]. 

Since fibroblasts express a similar genetic composition to that of neuronal cells, metabolite profiling of dermal fibroblasts of sporadic ALS has been carried out to assess the bioenergetic alterations.Gene expression of Sxc- was significantly reduced and glutathione peroxidase 6 (GPX6) was elevated in a cohort of patients characterized with hypermetabolism and trans-sulphuration pathways. In addition, the fibroblasts of this cohort have positively responded to anti-oxidant therapies under conditions of oxidative stress [143]. Altogether, under an oxidant environment, xCT is upregulated, causing an increase in the extracellular glutamate levels that induce Ca^2+^ mediated excitotoxicity. Although there is evidence for the upregulation of xCT—a functional subunit of Sxc-—in ALS mouse models and postmortem spinal cords of ALS patients, there are few discrepancies concerning their location. In the genetic mouse model of ALS, xCT levels were significantly found upregulated in spinal cord microglial cells, whereas it was specifically expressed only in astrocytes in human ALS postmortem spinal tissues [141,142]. This differential expression could be due to differences in species, for example, humans versus mice. Besides, the mouse study showed an increase in xCT gene expression whereas the human study demonstrates immunohistochemical localization in astrocytes.

xCT was also shown to be upregulated in animal models as well as in AD patients. As discussed above, oxidative stress ensures the overexpression of Sxc- which mediates glutamate-mediated excitotoxicity. Accordingly, Ashraf et al. found upregulated xCT levels associated with iron-dependent oxidative stress in the medial temporal gyri of AD patients. The study demonstrates an increase in the expression of iron-storage proteins, indicating the elevated labile iron levels in these patients, along with dysfunction of ferritin. A decrease in GPX4 (anti-oxidant system) and augmented lipid peroxidation was also observed, which explains the elevated xCT levels [144]. Previous evidence also showed that acute toxic insults, such as Aβ (1–42) peptide, 6-hydroxy dopamine (6-OHDA) and chronic AD conditions, increases levels of eukaryotic translation initiation factor eIF2α, to combat oxidative insult through xCT upregulation and thereby maintaining glutathione pools [145].

Similarly, Sxc- is implicated in PD. Upregulation of striatal Sxc- has been observed in several animal models of PD [134,146,147]. Genetic deletion of xCT prevented the dopaminergic neurodegeneration in substantia nigra pars compacta (SNpc) through the reduction in striatal glutamate levels in the 6-OHDA rat model of PD in both young as well as aged mice [134]. In a recent study, xCT deletion did not show any effect over 1-methyl-4-phenyl-1,2,3,6-tetrahydropyridine (MPTP) induced neurodegeneration [146]. On the other hand, xCT deletion conferred protection over Lactacystin-induced neurodegeneration in aged but not young mice [148]. Lactacystin is a proteasome inhibitor and induces PD differently compared with the other neurotoxins such as MPTP. It is reported that proteasomal inhibition induces xCT overexpression [147], which contributes to elevated extrasynaptic glutamate and thereby NMDAR activation induced neurotoxicity. The levels of xCT were found to be similar in both young and aged mice; however, deletion of xCT showed a protective effect against Lactacystin-induced neurodegeneration in old mice only. Altogether, these studies indicate a possible age-dependent association of xCT with proteasome degradation and neurotoxicity in PD [148]. 

A recent blood-based methylome-wide association study showed that hypermethylation of cg06690548 is linked to SLC7A11 (gene for xCT) downregulation, which causes glutathione depletion, oxidative stress, and dopaminergic neurodegeneration. The cg06690548 hypermethylation and the SLC7A11 downregulation are again observed to be associated with neurotoxin environmental exposure. This is supported by the fact that β-methylamino-L-alanine (BMAA) competes with Sxc- for cystine and causes glutathione depletion as well as glutamate release. BMAA, a neurotoxin found in seafood, is shown to cause neurofibrillary tangles and Aβ deposition [149]. These studies provide a strong evidence over the involvement of Sxc- in neurodegenerative diseases and poses Sxc- to be an attractive target to prevent the glutamate mediated excitotoxicity.

### 2.4. Hemichannels

Connexins (Cx) and pannexins (Pn) are two membrane protein families that form hemichannels, which are hexameric plasma membrane channels. Although these proteins do not have a fundamental structure that is comparable, their secondary and tertiary structures are similar. They have four helical transmembrane domains joined by one cytoplasmic and two extracellular loops, as well as intracellular N- and C- terminals [150]. Connexons or gap junction hemichannels are made up of two aligned connexin hexamers, one in each of the opposing membranes. Gap junctions (GJs) are a type of cell junction that allow molecules and ions to move between cells. They can exchange toxic or oxidative substances, such as excitatory amino acids, with neighboring cells and promote Ca^2+^ overload [151,152]. GJs are made up of hemichannels that are present on the cell membrane and, in conjunction with neighboring cells, they generate channels that allow GJ-mediated intercellular communication (GJIC). This allows for coordinated information flow, as well as metabolic substrate exchange and ion balance, between nearby astrocytes [153]. In the CNS, GJs are widely expressed in astrocytes where they couple these cells to create a functioning syncytium [154]. Importantly, hemichannel opening permits the release of glutamate [155]. Glutamate and other excitatory amino acids induce inflammatory responses in microglia, dendritic cells, and other antigen-presenting cells [156]. While a variety of clinical circumstances cause glutamate to be released by microglia and astrocytes via GJs, [157] glutamate is eliminated mostly by glial cells via EAAT1 and EAAT2 and it is rapidly recycled in the glutamate/glutamine metabolic cycle [158]. As a result, glial cells are not only in charge of safeguarding neurons from the negative consequences of high glutamate levels, but they are also an important source of glutamate clearance from the synapses [159]. Zu-Cheng et al. showed that, given glutamate’s functional importance, it is crucial to understand its travel and the control via the hemichannels. They reported in an in vitro system that astrocytes have functional hemichannels that can drive substantial glutamate and aspartate efflux [154]. NMDA receptors from the same or nearby astrocytes respond to these hemichannel-induced glutamate release, thereby causing Ca^2+^_alterations [160]. This glutamate released through hemichannels is required for NMDAR-dependent synaptic plasticity [161]. Connexin 30 (Cx30) and Cx43 are expressed by astrocytic cells, whereas Cx47, Cx32, and Cx29 are expressed by oligodendrocytes [162]. Individual Cx molecules form hexamers around a central pore to create connexons, also known as hemichannels, which are transmembrane channels. Interestingly, the hemichannels are not closed at rest while their likelihood of opening is very low but not zero. Cx43 is a Cx protein abundantly expressed in astrocytes and is found in gap junctions and hemichannels [163]. As a result, the activation of astrocytic Cx43 hemichannels is crucial for the ions to diffuse into the extracellular space from astrocytes, and also the release of ATP and gliotransmitters such as glutathione, adenosine, and glutamate [164]. According to recent studies, hemichannel opening at rest is crucial in basal synaptic transmission and long-term potentiation (LTP) [165,166]. Gliotransmitters are essential in the regulation of LTP. The hippocampal spatial short-term memory is aided by Cx43 hemichannels. Cx43 hemichannels permit the outflow of tiny molecules from astrocytes under specific pathological situations [153,166]. Higher instances of astrocytic hemichannels opening and reductions in gap junction interaction are linked to greater neuronal susceptibility and neuronal cell death in diseased situations [167]. Cx43, which is located on the mitochondria and astrocytic cell membrane, promotes neuronal damage. Orellana et al. found a novel mechanism involving the contribution of inflammatory glial cell signaling in neuronal death. When activated by Aβ, microglia produce pro-inflammatory cytokines such as TNF-α and Interleukin-1β, which enhance hemichannel activity in astrocytes. Subsequently, the opening of neuronal Panx1 hemichannels caused by the release of glutamate and ATP via Cx43 hemichannels could lead to neuronal death [168,169].

Nagy et al. reported that Cx43 expression was higher in reactive astrocytes surrounding amyloid deposition, hyperactivated microglia, and neurons in a classic AD investigation performed in human brains [170]. In cultured astrocytes and acute hippocampal slices, the Aβ peptide-induced hemichannel opening caused neuronal death via the release of glutamate and ATP [168]. In APP/PS1 animals, Cx43 and Cx30 expression was significantly increased in reactive astrocytes surrounded by Aβ plaques, along with enhanced Cx43 hemichannel activity demonstrated in acute hippocampal slices [171]. In the astrocytes of APP/PS1 mice, the elimination of Cx43 improved cognitive impairment [172]. Furthermore, in APP/PS1 animals, the deletion of astrocytic Cx43 blocks hemichannel activation and reduces neuronal injury in the hippocampus [171].

In the MPTP-induced PD animal model, which causes dopaminergic neurodegeneration, the striatal expression of Cx43 and Cx30 was found to be raised followed by an increased intracellular Ca^2+^ level in the astrocytes [173,174]. Furthermore, in the rotenone-induced model of PD, rotenone treatment caused the elevation of the Cx43 protein and thereby promoted its phosphorylation in both in vivo and in vitro studies [175]. Studies have shown how α-synuclein influences the function of astrocytic hemichannels. The opening of Cx43 and Panx1 hemichannels in the mice cortical astrocytes by α-synuclein causes changes in intracellular Ca^2+^ levels, nitric oxide generation, gliotransmitter release, mitochondrial structure, and survival capabilities of astrocytes [176]. Increased Cx43 expression has also been found in human brain samples diagnosed with AD and PD, a process that coincides with the course of both disorders [152].

It has been shown that oligodendrocytic and astrocytic GJ Cx43 proteins in the anterior horns of the spinal cords of mSOD1-Tg mice were significantly impacted at the disease-progression and end phases, suggesting that disruption of GJs among glial cells may aggravate motor neuron death and contribute to ALS [177]. Through hemichannels, Keller et al. further revealed the intimate interactions between activated microglia and astrocytes in late-stage ALS [178]. Another pathway in the SOD1-G93A mouse model of ALS indicates that astrocyte-mediated toxicity in ALS is an aberrant increase in Cx43 expression. Moreover, Cx43 levels were also found to be higher in the motor cortex and spinal cord of ALS patients. Therefore, neuroprotection through Cx43 blockers and Cx43 hemichannel blockers seemed beneficial [179]. 

### 2.5. Bestrophin-1 (Best-1)

Best1 is an anionic channel activated by Ca^2+^ that has a role in cellular activities such as maintaining Ca^2+^ homeostasis, the release of neurotransmitters, and regulating cell volume [180]. It is located in astrocytes residing in the cortex and hippocampus, glial cells of the cerebrum, reticular neurons located in the thalamus, meninges, and the choroid plexus epithelial lining. The striking feature of Best-1 is its ability to allow transport of large organic anions such as glutamate, GABA, and chloride ions. It is shown that, in hippocampal slice cultures, any stimulus under physiological conditions that increases the astrocytic intracellular Ca^2+^ concentrations induces BEST-1 mediated glutamate release at the microdomains. This released glutamate activates NMDAR and potentiates synaptic responses, modulating synaptic plasticity [181]. 

The structural changes in astrocytes during CNS insult and pathological conditions such as AD, serve to limit the injured area from spreading to other areas by barrier formation and preventing the immune cell infiltration and entry of other harmful substances [182,183]. Several reactive astrocytes surrounding the Aβ plaques have been found in AD patients [184]. The astrocytes also undergo a phenotypic switch from glutamate-producing normal astrocytes to GABA producing reactive astrocytes. Along with that, these reactive astrocytes show redistribution of Best-1 channels from perisynaptic microdomains to soma and processes and begin producing GABA tonically. This directs its focus from synaptic NMDA receptors to extrasynaptic GABA receptors. The tonically released GABA can negatively affect the synaptic transmission, plasticity and memory by inhibiting dentate granule cell excitability [185]. This could be one of the many plausible reasons for memory impairment in AD patients apart from neuronal cell death. 

Similarly, a substantial number of reactive astrocytes were also described in the SNpc of PD patients. It is possible that GABA released from these reactive astrocytes, via BEST-1, can reduce dopamine neuronal excitability and output. A study by Heo et al. demonstrates that the GABA released from reactive astrocytes reduces dopamine release in the nigrostriatal pathway by tonic inhibition and dopamine synthesis by downregulating tyrosine hydroxylase expression [186].

### 2.6. TREK-1

The TWIK related potassium channel, TREK-1, also called KCNK2 or K_2P_2.1, is a type of K_2P_ channel with a double-pore-domain background potassium (K^+^) channel. In CNS, it is mostly present in the GABAergic neurons and regions such as the basal ganglia, hippocampus, hypothalamus, and olfactory bulb. The channel activity is operated through various physical and chemical stimuli including stretching, cell swelling, temperature, polyunsaturated fatty acids (PUFA), and so forth. Mechanical stimuli such as stretching are directly transmitted through the lipid bilayer and cause the direct opening of TREK-1 channels [187]. 

Trek-1 seems to have several important physio-pathological functional roles in the CNS, mainly attributed to its potassium conductance and widespread presence. The function of neuronal TREK-1 in depression, pain, and ischemia has been explored [187]. In astrocytes, TREK-1 controls cell excitability by maintaining the membrane negative potential [188]. Upon heterodimerization, it mediates the passive potassium conductance and release of glutamate in astrocytes [189]. Based on their location on astrocytes, they can influence synaptic transmission. Mostly, they were found in the astrocytic soma and processes instead of perisynaptic domains, limiting their influence only to mGluR. Unlike Best-1, Trek-1 produces a rapid release of astrocytic glutamate resulting in fast inward currents in neurons through mGluRs [41]. Glutamate released by the astrocytes through TREK-1 acts on postsynaptic mGluRs and generates inward currents in the neurons [190]. Besides, cannabinoid, GABA_B_, adenosine A1, and opioid receptor activation cause Gi-GPCRs activation which is also shown to cause fast astrocytic Ca^2+^-independent glutamate release through TREK-1 stimulation in primary astrocyte cell cultures [191]. However, the physiological and pathological relevance of this glutamate release remains unclear. Owing to the colocalization of TREK-1 with opioid receptors, and opioid-induced glutamate release, they might contribute to the progression of addiction-associated behaviors [192].

Recent studies indicate that the TREK-1 confers neuroprotection through PUFAs as well as lysophospholipids against epilepsy and ischemia [187]. A very recent study in SAMP8, an accelerated aging model of mice, suggested the role of TREK-1 in AD pathology and learning deficits. In this study, TREK-1 activation through linolenic acid improved learning and memory by improving glutamate metabolism [193]. However, how exactly the TREK-1 contributes to learning and memory remains unexplored.

Riluzole, a non-specific TREK-1 activator, demonstrated protective effects in animal models of PD [193,194]. However, the principal effects of riluzole, such as NMDA antagonism in its neuroprotective efficacy, cannot be ruled out. In another study, low-intensity pulsed ultrasound improved neurodegeneration in the MPP+ model of PD by K2P channel activation [194].

### 2.7. Volume Regulated Anion Channels (VRACs)

VRACs are widely expressed in mammals and regulate the cell volume characteristics in physiological and pathogenic conditions linked to neuronal damage [195]. The astrocytic VRAC is stimulated by cell swelling and releases glutamate, thus reducing cell volume [196,197]. This massive glutamate release from swollen astrocytes mediated by VRAC activation overstimulates glutamate receptors in the neurons and induces excitotoxicity-induced neuronal death [198]. Neurological conditions such as epilepsy, ischemia and traumatic brain damage include swollen astrocytes as a characteristic pathological feature [199]. Interestingly, ATP modulates VRAC functioning via Ca^2+^ dependent cascades [200]. Under isotonic circumstances, this activated VRAC mediates glutamate release, suggesting that this channel may also have a physiological function [196].

SWELL1 (LRRC8A) is a widely expressed transmembrane protein with numerous leucine-rich repeats and is a key component of the VRAC channel. It is found in the plasma membrane where, knocking it out causes endogenous VRAC currents and a reduction in regulatory cell volume in a variety of cell types. Furthermore, point mutations in SWELL1 produce a considerable shift in VRAC anion selectivity [201]. This protein modulates synaptic transmission and neuronal excitability in astrocytes where VRAC functions as a glutamate-permeable channel in the presence of SWELL1 and facilitates glutamate release through tonic and cell swelling mediated mechanisms [202]. Cellular swelling in a high proportion of mammalian cells occurs via an increase in swelling-activated Cl^−^ currents, which are hypothesized to be associated with apoptosis, the modulation of membrane potential, and the secretion of physiologically active chemicals in addition to cell volume management [203].

The VRACs have been implicated in promoting inflammation. The cell swelling mediated by VRACs is known to alter cell volumes which triggers the activation of NLRP3 -inflammasome to cause inflammation [204]. Their activity is upregulated in the presence of ROS [205]. It is evident that the volume of excitatory amino acids release is controlled by the VRACs [206]. Recently, another report showed the ability of hydrogen peroxide to promote the activation of astrocytic VRACs followed by the release of excitatory amino acids [207]. The swelling of astrocytes in conditions of spreading depression was observed which released excessive glutamate through the VRACs [208]. Such swelling in astrocytes also prevented the conversion of glutamate to glutamine through the inhibition of glutamine synthetase, ultimately causing glutamate toxicity [209]. 

VRAC inhibitors could help in targeting this glutamate excitotoxicity [210]. Benfenati et al. reported, that carbenoxolone prevents volume-regulated anion conductance in cultured rat cortical astroglia [211]. Neuronal cell death was reduced by 80–95% when VRAC blockers were used [212]. 

As molecular identification is awaited, the evidence confirming VRAC as a channel of astrocyte that releases glutamate is mostly indirect and relies on nonspecific pharmacological inhibitors, impacting the activity of other membrane proteins, along with those engaged in the transportation of glutamate [203]. Therefore, even though the exact role of VRACs in neurodegenerative diseases like AD and PD may not be well elucidated, the glutamate excitotoxicity induced by them, in the presence of pathologies like Aβ oligomer and α-synuclein might prove to be detrimental in these conditions.

### 2.8. Purinergic Receptors

Another important mechanism for the release of glutamate from astrocytes is through the P2X Purinoreceptor 7 (P2X7) receptors. The P2X7 mediates the exocytosis of glutamate from the astrocytes [213]. Although the glutamate-specific channel opened by P2X7 ligand binding is not particularly selective, the considerable driving force for glutamate release in comparison to other anions promotes a significant glutamate efflux through these activated channels [214]. Ca^2+^ independent glutamate outflow from astrocytes has also been linked to P2X7 receptor-gated channels [214]. P2X7 receptor activation reduces glutamate uptake and glutamine synthetase activity in astrocytes through different pathways. The hypothesis that ATP could trigger glutamate release from astrocytes by binding to P2X7 receptors and driving channel opening was tested using mouse cerebral astrocyte cultures [214]. Both ATP and glutamate are bound by P2X7 receptors. In cultured and in situ astrocytes, activating these receptors causes ATP uptake and glutamate release at the same time [215]. Radiolabeled tracers were used to establish the release of L-glutamate and D-aspartate through P2X7 channels [214]. Furthermore, the Aβ 25–35 fragment causes intracellular Ca^2+^ concentration changes in astrocytes via connexin hemichannel opening and purinergic receptor activation, causing both Ca^2+^-dependent and independent glutamate release in the brains of the hAPP-J20 AD animal model [68].

Similarly, the activation of purinergic P2Y1 receptors was shown to release glutamate through Ca^2+^ mediated mechanisms [216]. Therefore, the role of purinergic receptors in glutamatergic transmission is crucial and needs further studies to understand the exact mode of action.

## 3. Conclusions and Future Directions

Current evidence proposes that the astrocytes play a crucial role in regulating the synaptic levels of glutamate, by mediating both uptake and release and thereby controlling the synaptic plasticity. Astrocytes exhibit various modes of glutamate release, each with a unique mechanism, specific location, time scale and selective neuronal target receptor, which ultimately influence the neuronal excitability. The astrocytic Ca^2+^-mediated vesicular exocytosis is a crucial mechanism that increases the synaptic pool of glutamate in various neurodegenerative diseases, although Ca^2+^-independent mechanisms cannot be ignored. The V-ATPases are functionally important for the vesicular release of glutamate and their deregulation is implicated in neurodegenerative diseases. Secondly, non-vesicular glutamate release by Sxc- has been well studied and implicated in various pathological states through extrasynaptic neuromodulation. The hemichannels—BEST-1, TREK-1 and VRAC—also facilitate effective glutamatergic transmission. However, their physiological and pathological contributions have not been fully understood and remain an area of extensive research. There are still a few major concerns to be resolved: what is the contribution of the vesicular and non-vesicular exocytosis of glutamate to the development of cognitive abnormalities in neurodegenerative disorders? In reactive astrocytes, what is the role played by the channels regulating the release of glutamate? Understanding these mechanisms might help us to control excessive glutamate release in the synaptic cleft that contributes to glutamate excitotoxicity. Therefore, striking a balance in the release and utilization of glutamate could be the key to managing these diseases for which further research is awaited. Owing to the advancements in technology, such as electrophysiological and optogenetic approaches, the presence of various channels and receptors involved in glutamatergic signaling over astrocytes is gradually exposed and rapidly emerging. Though the current evidence strongly indicates the release of astrocytic glutamate, their physiological significance and contribution to the development of neurological diseases are not yet fully known and remain to be explored. Understanding these mechanisms could help us shift our neurocentric approach to the role of astrocyte in physiological and pathological conditions and may be a promising therapeutic target for treating neurological disorders.
